# An exploratory study of delayed flash visual evoked potential P2 wave latency in subcortical arteriosclerotic encephalopathy

**DOI:** 10.1186/s12883-023-03388-z

**Published:** 2023-10-02

**Authors:** Lei Duan, Yu Ding, Gao-hui Sun, Yun-tao Li

**Affiliations:** 1 Department of Encephalopathy, Nanjing Jiangning Hospital of Traditional Chinese Medicine, Nanjing, 211100 China; 2 Department of Neurology, the Second Affiliated Hospital of Nanjing Medical University, Nanjing, 210011 China; 3 Department of General Medicine, the Second Affiliated Hospital of Nanjing Medical University, Nanjing, 210011 China

**Keywords:** Flash visual evoked potential P2 wave latency, MMSE, Subcortical arteriosclerotic encephalopathy, Cognitive dysfunction

## Abstract

**Background:**

Patients with cognitive dysfunction may present with significantly prolonged the P2 wave latency of flash visual evoked potential. However, no studies have been reported on whether the P2 wave latency of flash visual evoked potential is prolonged in patients with subcortical arteriosclerotic encephalopathy (SAE).

**Objective:**

To examine the relationship between flash visual evoked potential P2 wave latency (FVEP-P2 wave latency) and cognitive impairment in patients with SAE.

**Methods:**

Overall, we recruited 38 SAE patients as the observation cohort (OC) and 34 healthy volunteers as the control cohort (CC). We measured the FVEP-P2 wave latency for both groups. The SAE patients’ cognitive abilities were evaluated via mini-mental state examination (MMSE) and the association between the latency of FVEP-P2 and MMSE score was explored by Pearsons´s correlation test.

**Results:**

There is no significant difference between OC (21 males and 17 females; 68.6 ± 6.7 years of age and 9.6 ± 2.8 years of education) and CC (19 males and 15 females; 65.3 ± 5.9 years of age and 10.1 ± 2.6 years of education) in gender and age composition and education level. The FVEP-P2 wave latency of the CC group was (108.80 ± 16.70) ms and the OC FVEP-P2 wave latency was (152.31 ± 20.70) ms. The OC FVEP-P2 wave latency was significantly longer than the CC (*P* < 0.05). In terms of MMSE scores, the MMSE scores of CC was (28.41 ± 2.34), and that of OC was (9.08 ± 4.39). Compared to the CC, the OC MMSE score was significantly lower (*P* < 0.05). In addition, the FVEP-P2 wave latency was inversely related to the MMSE (*r* = -0.4465, *P* < 0.05) in SAE patients.

**Conclusion:**

The FVEP-P2 wave latency wave latency was significantly prolonged in SAE patients and strongly associated with the degree of cognitive dysfunction.

**Supplementary Information:**

The online version contains supplementary material available at 10.1186/s12883-023-03388-z.

## Introduction

Subcortical Arteriosclerotic Encephalopathy (SAE) is a special category of ischemic cerebrovascular disease, also known as Binswanger’s disease, associated with leukoaraiosis on neuroimaging and clinical symptoms of dementia in patients over 60 years of age with a history of hypertension and other atherosclerotic diseases [1–3]. The disease is common but rarely recognized because of the atypical symptoms, and there are many cases where SAE has been found at autopsy [4]. The disease is progressive and chronic, with cognitive and physical deterioration as the disease progresses, with cognitive dysfunction as the first symptom, especially memory loss and depressive symptoms [5].

At present, mini-mental state examination (MMSE), compiled by Folstein et al., is the most widely used neuropsychological scale to evaluate the mental state of patients [6]. Studies show that the cognitive function of normal elderly people will be affected by their age, gender and education level [7]. There are reasons to be skeptical that MMSE scores may be affected by the demographic characteristics of the test subjects Objective electrophysiological testing, including visually evoked potentials (VEPs) present more sensitive parameters that can be applied as indicators to hasten the detection of neurological pathological changes [8–11]. Of note, some authors described prolonged latency of the second positive component (P2) of the flash VEP (FVEP-P2) in amnestic mild cognitive impairment [11] and Alzheimer’s disease [12–20]. However, few studies explored FVEP-P2 in SAE properly.

As so, in the current study, we examined the P2 wave latency of FVEPs in SAE patients and elucidated the relationships between P2 wave latency and MMSE score, aiming to provide more robust clinical support for objective assessment of the mental state in SAE patients.

## Materials and methods

### Subject clinical information

This is a cross-sectional observational study of FVEP and MMSE responses from a group of SAE patients and healthy volunteers. Overall, 38 patients with SAE, who were admitted to the Department of Neurology in Nanjing Jiangning Hospital of Traditional Chinese Medicine, between January 2019 and March 2022, were recruited as the observation cohort (OC). In addition, we also recruited 34 healthy volunteers, who received physical examinations in our hospital during the aforementioned time period, as the control cohort (CC). This study was approved by the Ethics Committee of Nanjing Jiangning Hospital of Traditional Chinese Medicine (20,201,103). Informed consent was obtained from all individual participants included in the study.

### Inclusion and exclusion criteria

SAE patients and Healthy volunteers, from both genders and aged from 60 to 80 years old, were included. Subjects presenting other causes of dementia, such as Alzheimer’s disease and dementia with Lewy body, history of acute stroke, renal insufficiency, severe diabetes mellitus, congestive heart failure, uncontrolled hypertension within the past 3 months, cancer, schizophrenia, depression (score > 17 on the Hamilton depression scale); and refusal to sign informed consent [21] were excluded.

### The SAE diagnosis is based on the NINDS-AIREN Criteria

The NINDS-AIREN criteria [22] were: (1) Dementia was confirmed via clinical and neuropsychological examinations, and other brain-, systemic-, and Alzheimer’s-related diseases were eliminated; (2) a minimum of two of the following conditions were met: (a) risk factors for vascular or systemic vascular diseases (such as, hypertension, diabetes, prior myocardial infarction, arrhythmia, congestive heart failure); (b) diagnosed focal cerebrovascular disease (pyramidal sign or paresthesia); (c) apparent subcortical brain dysfunction; and 3. presence of several or diffuse subcortical hyperintensity features on the weighted magnetic resonance-T2 (MR-T2) phase.

### The detection method of FVEP

FVEP was detected using a NIP-200 evoked potential meter (Chongqing Haiweikang Medical Instrument Co., Ltd.). Our light source was a neon yellow light, carrying a wavelength of (590 ± 5) nm, a flash stimulation frequency of 1.0 Hz, a flash pulse width of 2 ms, and a flash number of 60 times. The subject was placed supine with eyes closed. 8 mm silver disk sunflower electrodes were positioned approximately 1.5 cm above the occipital tuberosity, with the electrodes on either side separated by 3 cm. The reference electrode was positioned at the midline forehead hairline, and the inter-electrode impedance was less than 50 kΩ. The light emitting diode (LED) arrays were positioned in a pair of eyecups, with 20,000 cd/m^2^ brightness to induce stimulation. Lastly, the bilateral FVEP-P2 wave latencies were measured separately.

### Cognitive function evaluation

Cognitive function evaluation was conducted using the following strategy and terms: “Mini Mental State Examination (MMSE)” [23, 24]. The score was completed independently by two researchers and then averaged. An unauthorized version of the Chinese MMSE was used by the study team without permission. The MMSE is a copyrighted instrument and may not be used or reproduced in whole or in part, in any form or language, or by any means without written permission of PAR (www.parinc.com).

### Statistical analysis

Data analyses was performed using the SPASS19.0 software. Homogenous and normally distributed data are presented as mean ± standard deviation (SD), and were analyzed via independent samples t-test. Pearson correlation was used to determine the association between FVEP-P2 wave latency and MMSE scores.

## Results

### Analysis of Basic Data Between the Two Groups

SAE patients (OC) were 38 patients (21 males and 17 females), with an age mean equal to 68.6 ± 6.7 years old and education of (9.6 ± 2.8 years of education. Healthy volunteers (CC) CC included 34 patients (19 males and 15 females) with an age mean equal to 65.3 ± 5.9 years 10.1 ± 2.6 years of education. No marked differences in the gender, age composition ratios, and educational level between groups were observed. (Table 1).

**Table 1:** Baseline characteristics of all participants

Baseline data	Control group (n = 34)	Observation group (n = 38)	*P*
Age (years)	65.3 ± 5.9	68.6 ± 6.7	> 0.05
Male/Female	19/15	21/17	> 0.05
Years of education (years)	10.1 ± 2.6	9.6 ± 2.8	> 0.05

### Comparison of the FVEP-P2 wave latency, MMSE scores between the two groups

As shown in Table 2, the FVEP-P2 wave latency of CC group was 108.80 ± 16.70 ms. The OC FVEP-P2 wave latency was at 152.31 ± 20.70 ms, compared to the CC (*P* < 0.05). In terms of MMSE scores, the MMSE scores of the CC group was 28.41 ± 2.34, and that of the OC group was 9.08 ± 4.39. Compared to the CC group, the OC MMSE score was significantly lower (*P* < 0.05).

**Table 2:** Analysis of the FVEP-P2 wave latency and MMSE scores between the two groups

Index	Control group (n = 34)	Observation group (n = 38)
FVEP-P2 wave latency (ms)	108.80 ± 16.70	152.31 ± 20.70^#^
MMSE (point)	28.41 ± 2.34	9.08 ± 4.39^#^

FVEP-P2 wave latency, flash visual evoked potential P2 wave latency; MMSE, mini-mental state examination. # Compared with the control group, *P* < 0.05

### Correlations between the FVEP-P2 wave latency and MMSE scores

**Fig. 1:**
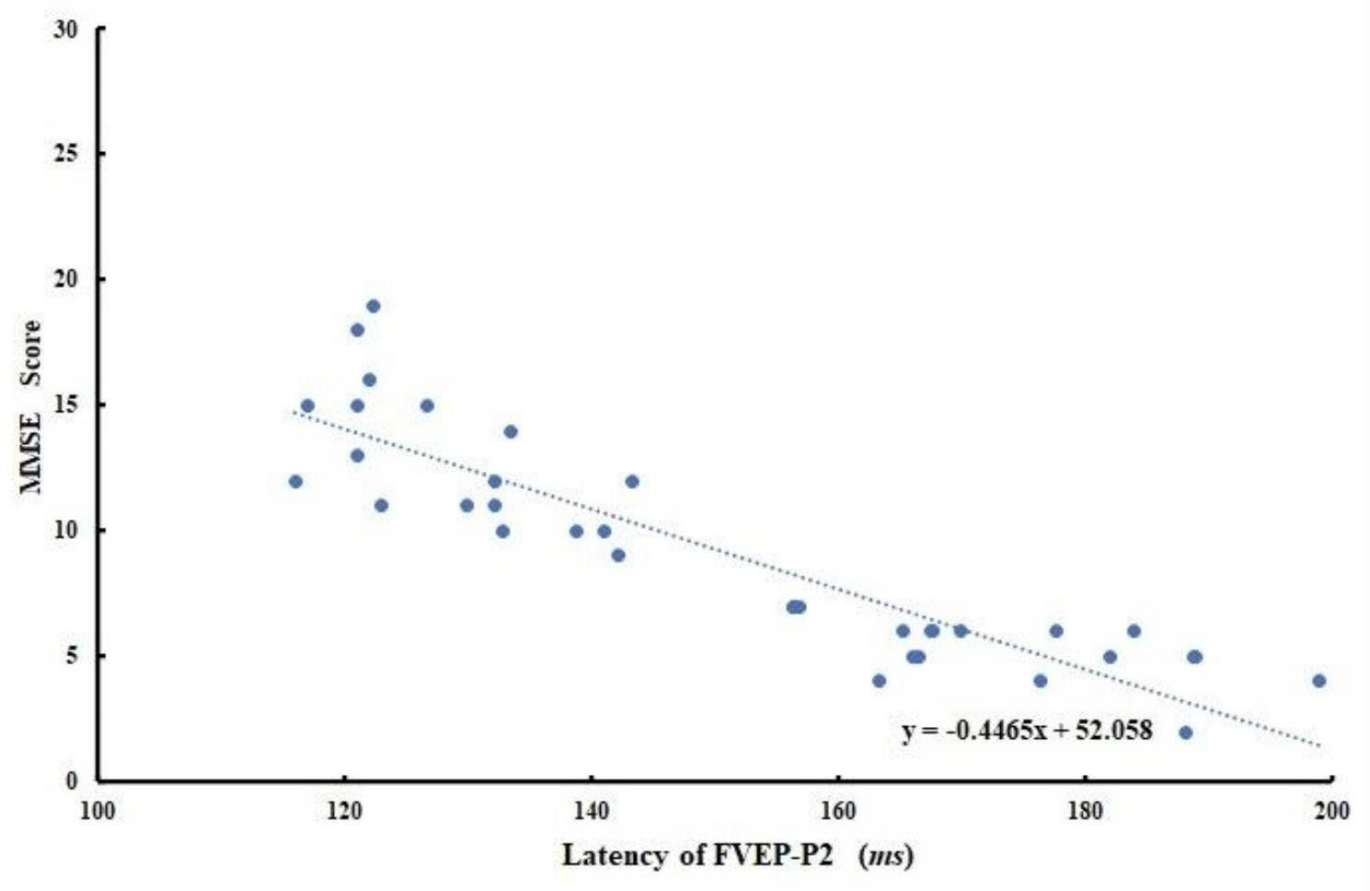
The negative association between flash visual evoked potential P2 (FVEP-P2 wave latency) latency and the mini-mental state examination (MMSE) score

Pearson correlation was used to determine the association between the FVEP-P2 wave latency and the MMSE scores, which was shown in Fig. 1. The FVEP-P2 wave latency was the abscissa; MMSE scores was the ordinate. With the growth of the FVEP-P2 wave latency, MMSE scores gradually decreased. The FVEP-P2 wave latency was negatively associated with MMSE scores (*r* = -0.4465, *P* = 0.007).

## Discussion

Herein, we retrospectively analyzed the FVEP-P2 wave latency and MMSE scores of patients diagnosed with SAE, in comparison to a control group of healthy volunteers, during hospitalization in our hospital. In this study, abnormal neural conduction in the visual cortex of SAE patients was clearly demonstrated on an electrophysiological basis. FVEP-P2 latencies were prolonged in relation to the control group and inversely related to the MMSE scores, which were drastically reduced. Limitations of this study are the small sample size and the missing information concerning current medications in use by SAE patients.

FVEPs can be separated into PVEPs and FVEPs. Studies have revealed that distinct mechanisms induce these two VEPs. The neural conduction network of PVEPs projects primarily through the lateral geniculate body of the occipital lobe to the middle temporal and parietal lobes and reflects macular function. In this case, the projection area is primarily the visual cortex. The FVEPs nerve conduction network initiates from the small retinal cells, and mostly projects to the visual area 4 and temporal lobe via the pretectal area, which is known as the association visual cortex [25–27]. Using human intracerebral recording technology, one study revealed that PVEPs originate in the primary visual cortex, while FVEPs originate in the striatum and extrastriate cortex [28, 29]. A study involving Alzheimer’s disease revealed a massive quantity of neurofibrillary tangles in the temporal lobe hippocampus, which primarily affects the visual cortex, and strongly regulates FVEPs, showing the prolonged FVEP-P2 wave latency, but no significant alterations were observed in PVEP-100 and latency [30].

The FVEP-P2 wave latency is the second positive-phase waveform in the FVEP. It has good stability and is closely related to the cholinergic nerve fibers in the brain’s visual cortex [31–33]. Studies revealed that anti-Parkinson’s anticholinergic drugs, but not dopamine, significantly prolong the FVEP-P2 wave latency. The above results indicate that the main neurotransmitter of association visual cortex nerve conduction is acetylcholine [34]. Acetylcholine concentration in the cerebrospinal fluid was reported to be significantly reduced in SAE patients as opposed to controls. Therefore, the prolonged FVEP-P2 wave latency in this study may be related to the reduction in acetylcholine levels, as well as the effect of cholinergic conduction [35, 36].

SAE is mainly characterized by subcortical white matter hyperintensity on MR imaging. Subcortical white matter is mostly composed of projection fibers that regulate cortical-cortical and cortical-subcortical nerve projection functions [37–39]. It has been reported the white matter structure is closely related to the latency and amplitude of the VEP waveform. [39, 40] Thus, we concluded that the prolonged FVEP-P2 latency in SAE may be related to leukoaraiosis, as well as to the abnormal function of projection fibers following a decrease in white matter density. However, its specific mechanism remains undetermined.

The present research has some limitations as follows. First, the sample size in this study was small. Second, we are missing information on the use (or non-use) of medications by patients, as some medications may cause a prolonged latency period for VEP. Finally, only the FVEP-P2 wave latency in SAE patients was found in this study, but the mechanism of FVEP-P2 wave latency in SAE patients has not yet been elucidated. In future studies, we will expand the sample size as much as possible and further elaborate the mechanism of FVEP-P2 wave latency in SAE patients.

## Conclusion

Prolonged latency of FVEP is strongly associated with the degree of cognitive dysfunction in SAE patients. Since reduced MMSE scores, subcortical leukoaraiosis, and prolonged FVEP-P2 latency seem to be closely related in this population, FVEP parameters may be considered as objective basis indexes of dementia status evaluating, from an electrophysiological point of view, and deserves a clinical promotion.

### Electronic supplementary material

Below is the link to the electronic supplementary material.

Supplementary Material 1

## Data Availability

The datasets generated and/or analysed during the current study are not publicly available due to privacy but are available from the corresponding author on reasonable request.

## Abbreviations

FVEP-P2: flash visual evoked potential P2
SAE: subcortical arteriosclerotic encephalopathy
OC: observation cohort
CC: control cohort
MMSE: mini-mental state examination
