# Supplementary material for: An exploratory study of delayed flash visual evoked potential P2 wave latency in subcortical arteriosclerotic encephalopathy
Source: BMC Neurol. 2023 Oct 2;23:345. doi: 10.1186/s12883-023-03388-z (PMC10544574; doi:10.1186/s12883-023-03388-z)
Supplement: Supplementary file 1 — Supplementary Material 1 [file 12883_2023_3388_MOESM1_ESM.pdf]

September 6, 2023

Yuntao Li
Department of General Medicine
The Second Affiliated Hospital of Nanjing Medical University
Nanjing, Jiangsu 210011
China

RE: Research titled, *An exploratory study of Delayed Flash Visual Evoked Potential P2 wave latency in Subcortical Arteriosclerotic Encephalopathy*

Dear Yuntao Li:

This letter is to confirm receipt of payment for your unauthorized use of an unauthorized version of the Chinese MMSE in the research study referenced above (Invoice IN-00244315).

Also by way of this letter, you may use the data obtained from said research to publish the results of this study, subject to the following conditions:

1) The publication **must not include** any test items from the unauthorized version of the Chinese MMSE that was used without permission, or any other version of the MMSE in any language.

2) The publication **must include** this statement:
An unauthorized version of the Chinese MMSE was used by the study team without permission.
The MMSE is a copyrighted instrument and may not be used or reproduced in whole or in part, in any form or language, or by any means without written permission of PAR (www.parinc.com).

3) One (1) copy of the article must be sent via email to PAR to confirm that the above conditions have been met.
Email: afernandez@parinc.com

If you have any questions or concerns, please do not hesitate to contact me directly.

Sincerely,

Andrea Butler Fernandez

Andrea Butler Fernandez
Jr. Permissions Specialist
afernandez@parinc.com
1-800-331-8378 (phone)
1-800-727-9329 (fax)
